# Cell therapy for severe burn wound healing

**DOI:** 10.1186/s41038-018-0117-0

**Published:** 2018-05-28

**Authors:** Zhe Li, Peter Maitz

**Affiliations:** 1 Burns Unit, Concord Hospital, Concord, New South Wales 2139 Australia; 2 Skin Laboratory, NSW Statewide Burns Service, Concord, New South Wales Australia; 3 Discipline of Surgery, University of Sydney Medical School, Camperdown, New South Wales Australia

**Keywords:** Skin, Burn injuries, Stem cells, Cell therapy, Wound healing, Skin regeneration

## Abstract

Cell therapy has emerged as an important component of life-saving procedures in treating burns. Over past decades, advances in stem cells and regenerative medicine have offered exciting opportunities of developing cell-based alternatives and demonstrated the potential and feasibility of various stem cells for burn wound healing. However, there are still scientific and technical issues that should be resolved to facilitate the full potential of the cellular devices. More evidence from large, randomly controlled trials is also needed to understand the clinical impact of cell therapy in burns. This article aims to provide an up-to-date review of the research development and clinical applications of cell therapies in burn wound healing and skin regeneration.

## Background

Burns remain one of the most traumatic injuries causing significant health, social and economic consequences [1]. Globally, severe burns lead to about 180,000 deaths annually and millions of patients suffering from non-fatal burns experiencing substantial and life-long physical and psychological morbidities.

Severe burn wound is characterised by the destruction of skin structures, functions and more importantly the loss of the progenitor cell populations that are essential for regenerating and restoring the structures and functions [2]. Until now, autologous skin grafting remains a standard practice in treating severe burns. However, its effectiveness is often challenged in treating severe burn patients with limited donor sites for skin graft harvesting. Consequently, the patients could experience a significant delay in wound closure, detrimental infection, scarring or even death.

To overcome the autograft shortage, a variety of alternatives for autologous skin grafts including allogeneic skin, xenografts and synthetic skin substitutes have been widely adopted in burn wound care [3, 4]. While those alternative devices provide temporary wound coverage and deliver various bio-factors to facilitate the angiogenesis and granulation of wound bed for further surgery, they could never replace the skin autograft delivering the essential autologous progenitor cells that could replicate to regenerate skin tissues for permanent wound closure. In past decades, cell-based therapies have emerged as popular choices in conjunction with standard skin grafting techniques for burn wound healing and regeneration of skin structure and functions. This article aims to provide an up-to-date review of the research development and clinical applications of cell therapies in severe burn wound healing.

## Review

### Development of cell therapy for burn wound healing

Cell therapy which also called cellular therapy or cytotherapy involves delivering an autologous or allogenic cellular component into a patient to repair or regenerate the damaged tissue due to injuries or diseases, to rectify the diseased conditions associated with the damages or deficiency of the unique cell population and to restore the physiological functions.

Skin as the multi-functional and protective barrier in human contains essential stem cell population and various cellular types that are critical for renewing and maintaining its structural integrity and functions. Research on skin cell transplantation for wound healing was first reported by Billingham and Reynolds in 1952 [5]. In a guinea pig model, they harvested both epidermal sheets and epidermal cell suspension by trypsin digestion and then transplanted them to surgically created wounds to evaluate their possible application in plastic surgery, experimental pathology, wound healing and contracture release. However, the era of cultured cell therapy for burn injuries was only opened up after Rheinwald and Green revolutionised the cell culture technique in 1975 allowing the isolation and serial cultivation of strains of human keratinocytes from a skin biopsy [6]. For the first time, epithelial sheets could be produced using cultured keratinocyte clones [7] in laboratory and cultured epithelia autografts (CEAs) were successfully transplanted in severe burns for wound healing [8]. The encouraging report further sparked worldwide research activities on cell therapy in burn research. Progresses as described in the following sections have been achieved over past decades to understand various cell types and their potential roles in burn wound healing.

### Different cell types with therapeutic potentials

Wound healing and restoration of skin structures and functions depend on many factors including the availability of essential progenitor cells, dermal extracellular matrix (ECM), and bio-factors and cytokines for angiogenesis and regulation of cell-matrix and cell-cell interactions. The following cell types have demonstrated potentials as therapeutic devices in skin wound healing and tissue regeneration. Many autologous and allogeneic cell products were developed using cells of skin and non-skin origins for therapeutic application in burn wound management.

#### Keratinocyte stem cells

The epidermis is mainly comprised of keratinocytes which are renewed and sustained by keratinocyte stem cell (KSC) populations anchored at the membrane in the epidermal-dermal junction, the hair follicle bulge and the sebaceous gland [9, 10].  KSCs, expressing K5, K14 and p63, are well known for regulating epithelial stratification, hair folliculogenesis and wound repair [10–12].

Based on molecular biomarkers, regenerative capability and the status of cellular differentiation, keratinocytes in human skin could be classified as KSCs, transient-amplifying keratinocytes and differentiated keratinocytes [13]. The KSCs, making about 4% to 8% of total keratinocyte population in skin [14], are localised at dermal-epidermal junction, hair follicles and the surrounding zones of skin appendages. They replicate themselves by asymmetric division to maintain a stable population of undifferentiated stem cells at the basal region and are essential for epidermal regeneration or repair due to skin injuries. Beta4 integrin is required for hemidesmosome formation, cell adhesion and cell survival [15]. During normal epidermal homeostasis or repair, the KSCs go through asymmetric division, exiting their niche, transforming into transient-amplifying keratinocytes and migrating towards skin surface to form multiple keratinocytes layers [11]. They gradually lose the capacity in population doublings through differentiation [10–15]. KSC populations with more active, proliferative and regenerative capacities are observed in young people comparing to aged peoples [16].

The keratinocytes can be easily isolated and expanded in numbers under in vitro cell culture conditions. Keratinocytes produce various bio-factors and cytokines including interleukin (IL)-1, IL-6, IL-7, IL-8, IL-10, IL-12, IL-15, IL-18, and IL-20 and tumour necrosis factor alpha (TNF-α) [17], which are important for regulating skin regeneration and wound healing. All these properties make them the most important and widely used cells for therapeutic application in burn wound care.

A population of circulating keratinocyte progenitor cells (KPCs) was identified with p63 in peripheral blood, which differentiated into keratinocytes with expression of the cytokeratins, involucrin and filaggrin [18].

#### Dermal fibroblasts

Dermal fibroblast cells play an important role in normal skin and skin wound healing post-burn. They produce the key ECM proteins in the dermis including laminins, fibronectins, collagens, elastic fibres, non-collagen molecules and bio-factors to regulate cell function, migration and the cell-matrix and cell-cell interactions in normal skin homeostasis and wound healing [19–21]. Dermal fibroblast cells produce many growth factors and cytokines including vascular endothelial growth factor (VEGF), basic fibroblast growth factor (bFGF), hepatocyte growth factor (HGF), platelet-derived growth factor (PDGF)-AA, transforming growth factor-beta1 (TGF-β1), keratinocyte growth factor (KGF), IL-6 and IL-8 and tissue inhibitors of metalloproteinases [19–21]. Cryopreserved cultured dermal substitute maintained sufficient fibroblast cell viability and the ability to proliferate and release a significant amount of VEGF [22]. Epidermal-derived pro-inflammatory cytokines IL-1α and TNF-α mediate synergistically the secretion of wound-healing mediators (with the exception of sST2) from fibroblasts in dermal substitutes. Studies demonstrated a synergistic enhancement of the expression of bio-factors including IL-1α, IL-6, bFGF, CCL2, CXCL1, CXCL8, and sST2 if KSCs and dermal fibroblast cells were co-cultured in a dermal regenerative scaffold [21, 23, 24].

Skin-derived precursor (SKP) cells have also been isolated from dermal papillae and can be differentiated into adipocytes, smooth myocytes and neurons in vitro [25, 26]. Fibroblasts from adult mouse and human can be induced into pluripotent stem cells with potentials for regeneration of various tissues [27, 28].

#### Mesenchymal stem cells

Mesenchymal stem cells (MSCs), a unique population of multipotent cells with self-renewal and regenerative capacity can be derived from autologous or allogenic tissues including bone marrow, adipose tissue, nerve tissue and umbilical cord and cord blood, and dermis. MSCs express surface CD markers including CD44+, CD73+, CD90+ and CD105+ and are distinguished from haematopoietic cells by a lack of CD34, CD14, CD45, CD11b/CD79 and CD19/HLA-DR [29–32].

MSCs produce and release bio-factors [33–37] such as VEGF, stromal cell-derived factor-1, epidermal growth factor, KGF, insulin-like growth factor and matrix metalloproteinase-9 promoting angiogenesis; recruit endogenous progenitor cells; and direct cell differentiation, proliferation and ECM formation during wound repair. MSCs also secrete cytokines including interferon-λ, TNF-α, IL-1α and IL-1β, and nitric oxide to modulate host immune response in wound healing [33, 36, 37].

Studies demonstrated that both autologous and allogenic MSCs, administered both systemically or and locally, exhibit therapeutic potentials promoting cutaneous wound healing and tissue regeneration via the paracrine bio-factors and cytokines and the multi-potency in tissue regeneration [31, 32, 38–40]. Under specific niche conditions and molecular stimulations, MSCs could be induced to differentiate into multiple tissue-specific cell lineages including osteoblasts, adipocytes, chondrocytes, tenocytes, myocytes, endothelial cells, vascular smooth muscle cells, keratinocytes and sweat gland-like structures [35, 37, 41]. MSCs could produce anti-fibrotic factors and modulate development of hypertrophic scarring [42]. Adipose-derived stem cells (ADSCs) and ADSC-conditioned medium appear highly effective in promoting hair growth [43, 44], and development of functional sweat gland-like structures was observed from transplanting differentiated bone marrow mesenchymal stem cells (BM-MSCs) [45]. MSCs promote angiogenesis and vascular stability that are critical for delivering the essential nutrient supply for tissue regeneration and wound healing [46].

#### Induced pluripotent stem cells

Human embryonic stem cells (hESCs), derived from human embryos are well known for their pluripotent capacities in tissue regeneration. Research demonstrated that hESCs could be induced to efficiently differentiate into functional basal keratinocytes that regenerate a pluristratified epidermis [47–51]. Immortalised keratinocyte lines [52] and fibroblasts [53] were also derived from hESCs. The hESCs-derived fibroblasts were observed to direct development and repair of three dimensional (3D) human skin equivalents [53].

Although hESCs could be developed to provide temporary skin substitutes for burn patients awaiting autologous grafts, the potential applications are however complicated due to the ethical issues in relation to using human embryos.

The Nobel Prize winning discovery of induced pluripotent stem cells (iPSCs) by Yamananka and his team brought an alternative source of stem cells with pluripotent potentials for therapeutic applications [27, 28]. While avoiding the ethical concerns in using hESCs, they induced adult mouse or human dermal fibroblasts into pluripotent stem cells by transforming them with Sox2, Oct4, Klf4 and c-Myc transcription factors using a retroviral system. The generated iPSCs are, although not identical, extremely similar to embryonic stem cells being capable of generating all cell lineages of different tissues including skin [49, 54]. iPSC-derived cells could secrete proteins including VEGF, FGF-2, TGF-β, S100A4, GRO, GM-CSF, MCP-1, IL-6 and IL-8 that promote increased proliferation, contraction and migration [55]. iPSCs as patient specific or allogenic devices demonstrated potential values for clinical applications, research and drug discoveries in laboratory research. Their safety and efficacy are still to be assessed mainly due to the use of retroviral vectors, associated risk, genetic instability and potential immunogenicity [56]. To avoid the risks of mutagenesis and oncogenic transformation associated with retroviral vector, iPSCs are also generated with non-integrative reprogramming strategies using plasmid vectors, episomal plasmid vectors, modified/microRNA or even direct delivery of reprogramming protein factors [57–59]. The non-integrative reprogramming strategies were considered safer for therapeutic purpose. However, the efficiency of iPSC induction by original plasmid vector was low although it could be enhanced by essential factor for episomal amplification of the vector [58].

### Clinical research and applications of cell-based therapies in burn wound care

#### Cultured epithelial autografts

The key technique that Rheinwald and Green established in the 1970s for serial culture of keratinocytes opened the era of research and therapeutic uses of cultured autologous keratinocytes in burn wound healing. Under laboratory conditions, KSCs could be isolated from a small skin biopsy and cultivated in the presence of growth-arrested 3T3 mouse fibroblasts as feeder cells to form epidermal sheets that could be transplanted back to the same patient for treatment. The innovative technology quickly gained global attention and emerged as a popular way to generate alternative autografts to facilitate permanent wound closure when treating severe burns. Since 1981, both cultured living keratinocytes and epithelial autograft sheets (CEAs), either produced in house or by commercial companies, have been used as clinical devices in treating large burns [8, 60–67].

Cultured keratinocytes were used for treating burns in various ways. Commonly, the cells from a skin biopsy are cultivated, screened and passaged in a cell culture system to expand the numbers of KPCs. In the presence of feeder fibroblast cells, the keratinocytes could proliferate and differentiate to form an epidermis-like tissue in cell culture. The process of CEA cultivation usually requires about 3 weeks depending on many factors including patient age, health condition and severity of burn injuries. The colony-forming efficiency of keratinocytes from freshly trypsinised skin was reported very low, between 0.15% and 3.8% in the primary cell culture [68–70] but was increased to 26% to 90% at passage 2 to 3 cultures and to over 16% in CEA sheets [70], being significantly higher than that (4–8%) in normal skin [14]. Cultured epidermis can be harvested as a sheet graft for clinical application.

Cultured human sole-derived keratinocyte grafts re-express site-specific differentiation after transplantation [71]. The formation of hair follicles and development of normal epidermal microarchitecture were observed when epidermal cells were transplanted together with cells of dermal origin [72].

Very often, CEAs were used in combination with widely meshed skin grafts for wound closure. CEAs could be transplanted directly to the wound area [8, 73] with undifferentiated progenitor cells attached to the regenerated or freshly debrided wound bed [74]. CEAs were of significant value in severe burn management, providing wound coverage, facilitating wound epithelisation and permanent closure, and improving survival rates among patients with massive burn wounds [75–78]. CEA demonstrated a very high beneficial value in managing burns > 60% total body surface area (TBSA), being a life-saving treatment [79]. Although being expensive compared to standard skin autograft, the engraftment rates and survival ratio results make CEAs excellent alternative wound coverage when donor sites are limited in severe burns [80]. Studies reported an average graft take of 72.7% with a 91% overall survival rate in treating critically burns using CEA, indicating cultured keratinocytes as an excellent alternative or adjunct to conventional split-thickness skin grafting [81, 82]. The CEAs were reported to enhance the take of a wide meshed autograft in massive burns and allow for grafting wide meshed autograft together with acellular dermal matrix in some cases [83] and were observed to improve skin texture of burn scars after resurfacing the wounds [84]. The use of CEA in treating a full-thickness skin wound in severely burned patients results in favourable quality of scars and was reported to show good potential to save lives by providing epidermal cover [85]. Early excision followed by temporary coverage with autograft, which is allowed to engraft, was found to be associated with a low infection rate and a higher rate of CEA take [86]. CEAs regenerate a stable normal epidermis and are capable of inducing dermal regeneration from wound bed connective tissue [68, 84]. Better outcomes in aesthetic appearances and scar formation were also reported in burn patients grafted with CEAs [87].

In addition, the procedure can be initiated with a small skin biopsy causing minimum donor site morbidity. This would be particularly important for patients with extensive burns as a life-saving device. CEAs deliver living autologous epidermal stem cells for permanent wound closure without eliciting immunological rejection. CEAs could also function as biological wound dressings delivering the much needed biological factors and niche environment for wound healing.

#### Cultured keratinocyte suspension

In 1952, Billingham and Reynolds examined the potentials of transplanting sheets of pure epidermal epithelium and non-cultured epidermal cell suspensions for wound healing in guinea pigs [5]. In treating burn patients, the burn wound areas often require early excisions and then immediate skin grafting. While a skin sample could be taken for growing CEAs, a 3-week waiting time for CEA growth would be too long for many patients as it unnecessarily delays the surgical intervention. Consequently, the keratinocytes were cultured only to sub-confluent stage or be grown on carrier material to allow early harvesting and application as cell suspension or sub-confluent sheet [88, 89].

Autologous keratinocytes isolated from any available donor site area including sole and scalp [90] could be isolated and cultured for clinical application. The delivery of cultured keratinocytes in suspensions, directly to the wound or in combination with skin grafting, accelerated epidermal wound healing in animal models and burn patients [90, 91]. The transplantation of cultured keratinocytes were found to facilitate the reformation of organised epidermal structure [69] and to promote permanent epithelialisation in the burn wound [92, 93] and accelerated epithelial maturation in an in vivo wound model [94]. Permanent repigmentation of piebaldism was also reported in patients treated by erbium:YAG laser and autologous cultured epidermis [95].

To ensure the effectiveness of living keratinocytes for burn wound healing, the cells were delivered in many ways. Co-spray of cultured keratinocytes and autologous fibrin sealant helped retaining the cells in wound and appeared an effective means of keratinocyte delivery for wound healing [96, 97]. Grafting of a fibrin-based culture improved adhesion and development of the epidermis and graft take onto the artificial dermis [98]. Cultured keratinocytes sped up the epithelisation when used in combination with the Meek technique in achieving wound closure in the severely burned paediatric patients [99] and led to permanent burn wound coverage when being grafted with allodermis in burn wound [100, 101].

Sub-confluent autologous keratinocytes were also grown on polymer materials and successfully transferred to achieve would closure [102–105]. The technique significantly reduced the grafting time to within 5–7 days of receipt of biopsy and ensured the cell delivery. High yields of proliferating autologous keratinocytes can be grown on gelatin microbeads and delivered to epithelialise cutaneous wound [106–108]. Less wound contraction was reported when keratinocytes on carrier beads were delivered for wound healing [107].

#### Non-cultured epidermal cell suspension

A quick procedure using non-cultured autologous epidermal cells for wound healing was also reported [5, 109]. It involved isolating the epidermal cells from a small skin sample by enzymatic digestion, preparing a basal cell-enriched suspension and redistributing the cells by spraying or dripping to the debrided or granulated wound area to facilitate permanent wound closure or to correct the pigmentation loss. The isolated living cells were then re-distributed immediately to a larger wound surface area at 1:80–100 expansion ratio for fast wound epithelisation [110].

The use of non-cultured epidermal suspension is a fast procedure that involves minimal tissue manipulation and can be completed within a couple of hours in surgical settings. The non-cultured epidermal cells can be used alone or in combination with meshed grafts for wound healing. It was reported to promote wound epithelialisation and reduce the required surgical intervention and total length of stay in treating burns and donor site wounds [109, 111]. However, wound re-epithelialisation and the number of keratinocyte colonies were significantly less in wounds transplanted with non-cultured keratinocytes compared to wounds with cultured keratinocytes [91].

Due to the existence of melanocytes in epidermal harvests, the non-cultured epidermal cell suspension helped in correcting the skin hypopigmentation post burns or repigmenting vitiligo [112–115].

### Scaffold-guided co-culture and skin regeneration

Severe burns usually involve detrimental damages to the dermal matrix niche that is essential for structural support, cell attachment, migration, proliferation and differentiation. While CEAs, cultured keratinocytes or epidermal cell suspensions facilitate wound healing and epithelialisation, they showed limitations in treating deep burn wounds that lack dermal foundation. Artificial dermal regenerative templates such as Integra and MatriDerm [4] are commonly used for dermal regeneration in treating deep burns. They are porous bio-scaffolding structures made of biodegradable polymers including proteins and proteoglycans. They provide structural supports and niche matrix for cell attachment, migration and proliferation, and more importantly, they allow the concurrent application of multiple cell types, cell-cell and cell-matrix interactions for engineered skin tissue regeneration and therapeutic purposes. Dermal scaffolds together with cultured keratinocytes and fibroblasts enable in vitro generation of living, durable skin substitutes within several weeks, with reconstructed epidermis and dermis of close to normal histological appearance [116, 117]. Scaffold-guided co-culture of different skin cells synergistically enhance the production of bio-factors and cytokines that benefit wound healing. Increased expression of integrins and decreased apoptosis correlate with increased melanocyte retention in cultured skin substitutes [118]. The autologous living skin substitutes were proved to be clinically effective in severe burn management, resulting in permanent wound closure with improved aesthetic outcomes and minimising donor site casualty [115]. In addition, other cell types including endothelial cells, melanocytes, MSCs could also be seeded to produce engineered skin equivalents with microvascular network, skin appendages and pigmentation, which share more structural and functional similarities to natural skin [119–122]. The in vitro pre-microvascularisation of engineered skin substitute could inosculate with host vasculature to enhance in vivo graft survival. 3D Skin Equivalents were also reconstituted from human iPSCs [54]. Co-culture of KSCs and dermal fibroblast cells with dermal regenerative scaffold enable the development of living skin substitute with structures comparable to natural skin. It indicated that the full-skin substitute has a greater potential to stimulate wound healing than epidermal or dermal substitutes alone.

#### MSC- and iPSC-based therapies

MSCs including BM-MSCs and ADSCs have demonstrated great potentials in acute and chronic wound healing and skin repair [123–125]. They promote wound healing through enhanced cell migration, differentiation and release of signalling cytokines and bio-factors to regulate the process of angiogenesis [126, 127].

The subcutaneous adipose tissue from debrided skin in burn surgery can be a rich source of ADSCs for many applications such as being grafted as cutaneous wound therapy or for tissue engineering [128]. Autologous ADSC-enhanced healing of cutaneous radiation syndrome was observed in a mini-pig model [129].

BM-MSCs were reported to enhance wound healing quality and facilitate skin regeneration after full-thickness injury, with the grafted cells transdifferentiating into cell types including vascular endothelial cells, sebaceous duct cells and epidermal cells [122].

Allogenic human umbilical cord mesenchymal stem cells (hUCMSCs) and BM-MSCs are being trialled clinically for treating acute or second-degree burns [130, 131] but no results have been reported until now. BM-MSCs in hydrogels could be a promising therapeutic strategy for curing chronic wound including diabetic ulcers [124]. Autogenic BM-MSCs transferred with collagen membrane were seen to repair full-thickness cutaneous deficiency in porcine model [132]. ADSCs were reported to modulate development of hypertrophic scarring in a red Duroc porcine model [42] and attenuate scar formation during wound healing [133, 134].

Patient-specific and gene-corrected iPSCs, expressing collagen 7, were developed with the inherent capacity to treat recessive dystrophic epidermolysis bullosa, a severe and often lethal condition caused by mutations in the COL7A1 gene-encoding type VII collagen [135]. Very recently, the entire human epidermis could be generated using transgenic stem cells for the genetic disease [136].

### Considerations and future directions

The evidence from laboratory, animal and clinical studies demonstrate that cell-based therapy is becoming an important alternative or conjunctive treatment for burn wound management. It is important to point out that cell therapy devices and associated procedures are now highly regulated by global authorities to ensure their bio-safety and efficacy. While animal sourced reagents could cause concerns, unresolved issues also exist in the potential applications using MSCs and iPSCs. It is also important to understand that MSCs from different sources could have different immunomodulation capabilities and varied capacities to proliferate and differentiate to various cells. If allogeneic MSCs are used, their immunogenicity could have impact on their in vivo durability. More studies are needed to define those issues as they could potentially affect the therapeutic outcomes [137]. Also, MSCs were reported to enhance tumour formation and growth in vivo and metastasis [138–143]. The biosafety of retroviral vectors for transcriptional factor delivery in making iPSCs also need to be examined in long-term investigations for any associated cancer risk, epigenetic memory retained from parent cells, genetic instability and potential immunogenicity [56, 144].

Until now, cultured keratinocytes and non-cultured epidermal isolates are the most used devices for treating burns. Cultured epidermis and skin substitutes are only structurally similar but will never be identical to natural skin as evidenced by the differences in their gene expression profiles [145]. They are fragile and more susceptible to mechanical shearing forces and are more sensitive to infections due to an insufficient level of beta-defensins, the antimicrobial peptides [146]. CEAs also displayed poor adherence, poor stability and delay in rete ridge formation and elastin filament development [147] and lacking or delayed basement membrane [117] post grafting. Although the potential and feasibility for the use in the treatment of burns were demonstrated, the data were mainly generated from laboratory analysis, animal studies, case reports and observations of small patient cohorts lacking of proper controls. More reliable and randomly controlled trials in a large scale are still needed to assess the efficacy and to understand the full capacities of each cellular device in burn wound healing, scarring and skin repair.

More research and development should be carried out to address the following technical problems and factors that could affect the outcomes of cell therapy. For example, the time needed for generating autologous CEAs or scaffold-guided living skin substitutes is often too long for managing acute burns. Technical improvement is needed to ensure the timely supply of cultured cellular products for burn wound healing when early debridement was carried out in treating burns. The use of cultured cell suspension as well as non-cultured cells significantly reduced the preparation time for cellular products but came across other issues following their delivery. The cloning efficiency is more of a concern with non-cultured epidermal cells as the freshly isolated cells showed very low efficiency in cloning or replicating themselves [63, 68, 70]. The run-off issue of cell suspension from the wound surface could further decrease the number of cells actually retained in the wound after delivery. In addition, other factors including wound depth, wound bed preparation, and wound infection further complicate the application of cell therapy and lead to variable outcomes. As timing is always critical for surgical intervention in treating burns, the successful use of cell therapy also depends on the well-coordinated collaboration between clinical scientists, surgeons and nursing staff.

## Conclusions

Cell therapy has emerged as an important component of life-saving and wound healing procedures in treating burns. Although progresses have been made to demonstrate the potential and feasibility of various stem cells for burn wound healing, there are still scientific and technical issues that should be resolved to facilitate the full potential of the cellular devices. More evidence is needed from research and large, randomly controlled trials to define the clinical efficacy and safety of cell therapy in burns.
